# Biodegradable Carrageenan-Based Force Sensor: An Experimental Approach

**DOI:** 10.3390/s23239423

**Published:** 2023-11-26

**Authors:** Uldis Žaimis, Jūratė Jolanta Petronienė, Andrius Dzedzickis, Vytautas Bučinskas

**Affiliations:** 1 Institute of Science and Innovative Technology, Liepaja University, 3401 Liepaja, Latvia; 2 Department of Mechatronics, Robotics, and Digital Manufacturing, Vilnius Gediminas Technical University, 10105 Vilnius, Lithuaniavytautas.bucinskas@vilniustech.lt (V.B.)

**Keywords:** force sensor, stretch sensor, force sensor, k-carrageenan, *Furcellaria lumbricalis*, iron (III) oxide, piezoresistivity

## Abstract

The development of low-cost biodegradable pressure or force sensors based on a carrageenan and iron (III) oxide mix is a promising way to foster the spread of green technologies in sensing applications. The proposed materials are inexpensive and abundant and are available in large quantities in nature. This paper presents the development and experimental study of carrageenan and iron (III)-oxide-based piezoresistive sensor prototypes and provides their main characteristics. The results show that glycerol is required to ensure the elasticity of the material and preserve the material from environmental impact. The composition of the carrageenan-based material containing 1.8% Fe_2_O_3_ and 18% glycerol is suitable for measuring the load in the range from 0 N to 500 N with a sensitivity of 0.355 kΩ/N when the active surface area of the sensor is 100 mm^2^. Developed sensors in the form of flexible film have square resistance dependence to the force/pressure, and due to the soft original material, they face the hysteresis effect and some plastic deformation effect in the initial use stages. This paper contains extensive reference analysis and found a firm background for a new sensor request. The research covers the electric and mechanical properties of the developed sensor and possible future applications.

## 1. Introduction

Flexible force or pressure sensors, known from 1900 [1], have currently become among the most demanded electronic components. The wide variety in their typical application fields set specific requirements for such sensors: they must be functional, inexpensive, meet environmental protection requirements, and be harmless to humans. Flexible force sensors are required to quantify the applied force or pressure, efficiently converting the mechanical deformation into an electrical signal [2]. The flexibility of the force sensor allows them to conform to irregular surfaces, allowing more accurate and precise force measurements [3]. Flexible force sensors typically consist of a flexible substrate material, such as polyimide, silicone, etc., that serves as the base for the sensing elements and conductive materials, such as carbon or silver particles [4]. The specific sensing mechanism may vary depending on the design, but common approaches include piezoresistive, capacitive, or optical sensing principles [5,6,7].

The operating range of force sensors generally depends on the properties of the material used and the mechanical design of the sensor [8]. A well-known example of such a sensor is a force-sensitive resistor (FSR) [9]. With the emergence of flexible force sensors, some of the FSR problems were solved, but new challenges were raised, and the best combination of substrate and conductive materials can still be improved. However, gel-based sensors typically suffer from significant hysteresis and sensor sensitivity variation with respect to time or changes in the environmental conditions. Therefore, the main challenge parameter for the piezoresistive flexible force sensors is repeatability and linearity, which could be ensured by improving the characteristics of the sensitive material or implementing smart signal acquisition [10] and processing techniques. Multi-criteria research on flexible force sensors leads to various unexpected solutions, such as the construction of flexible force sensors on a piece of paper [11].

The aim of this paper is to present the experimental research on piezoresistive flexible force sensors produced from the original combination of a carrageenan-based substrate and iron oxide conductive fillers. The paper presents the most promising combinations of material compositions and provides detailed characteristics of the sensor prototypes.

## 2. State-of-the-Art: Natural-Materials-Based Force Sensors

Biodegradable force sensors are a specific type of technology designed to be environmentally friendly and capable of naturally degrading over time. These sensors are typically made from biocompatible and biodegradable materials, allowing them to break down and dissolve into nontoxic components when exposed to specific environmental conditions. Flexible biodegradable force sensors find applications in various fields where temporary force monitoring or sensing is needed, for example, in biomedicine, environmental monitoring, or agricultural applications. Like most flexible sensors, they can also be used in medical implants or wearable devices to monitor the forces applied during rehabilitation or assistive activities [12,13]. The construction of biodegradable force sensors often involves the use of organic or biobased materials, such as natural polymers or composites. The materials for constructing biodegradable force sensors are usually selected by their ability to degrade through natural processes such as enzymatic or microbial degradation [14,15]. Crucial in the context of this work are studies of the aerobic composting of biodegradable plastics; therefore, a recent report by Kalita et al. [16] presents a mathematical model for the biodegradability of several polymers with good empirical coincidence.

### 2.1. Biodegradability of Biosensor Materials

It is worth noting that the research and development of biodegradable force sensors is ongoing, and advancements in materials science and sensor technology continue to improve their performance, sensitivity, and degradation properties. Reviewing the most exciting solutions for biodegradable polymer-based force sensors, the work of Yu et al. [17] is worth mentioning. Researchers have developed biodegradable force sensors using polymer-based materials, such as polylactic acid (PLA) or polyhydroxyalkanoates (PHA). These sensors can be integrated into medical implants or wearable devices to monitor the forces exerted during rehabilitation exercises or assistive activities. Ma et al. [18] presented another eco-friendly force sensor solution for temporary force monitoring based on the use of starch—a renewable and biodegradable material developed by incorporating conductive materials within a starch matrix.

The sustainable use of cellulose or cellulose-nanofiber-based materials for sensors is also an essential issue; for example, Su et al. [19] proposed that cellulose nanofibers derived from sustainable sources such as wood or plants could be used to produce biodegradable force sensors. These sensors exhibit good mechanical properties and can measure forces in various applications, including environmental monitoring or agricultural settings.

Silk-based force sensors are also considered a sustainable solution [20,21]. This natural protein fiber is suitable for the development of biodegradable force sensors, which can be implanted or integrated into medical devices to measure forces in biomedical applications. These sensors are biocompatible and gradually degrade with time.

Biodegradable gelatin, derived from collagen, has been investigated for creating a biodegradable force sensor by Wang et al. [22]. The authors defined that the incorporation of conductive materials or microstructures within gelatin matrices makes it possible to develop sensors to monitor forces in applications such as tissue engineering or drug delivery systems.

Generally, the use of biodegradable force sensors offers several advantages. First, they minimize environmental impact by reducing the accumulation of non-biodegradable waste. Second, they eliminate the need for sensor retrieval or removal after use, as they naturally degrade over time. This feature is particularly beneficial in applications where the sensors are implanted or deployed in hard-to-reach or sensitive environments. Finally, biodegradable sensors can provide real-time force data during their functional lifespan, enabling the monitoring and analysis of force-related parameters. 

### 2.2. Application of Seaweed-Derived Carrageenan in the Development of Biodegradable Force Sensors and Combination with Metal Salts and Metal Oxides

Red algae are photosynthetic eukaryotes with an extracellular matrix consisting of a complex of supramolecular networks connecting cells. The structure of the extracellular complex depends on the algae species and the life-cycle stage. The main components of the extracellular complex are sulfated galactans such as agars, porphyrins, and carrageenans [23]. Thus, polysaccharides retain water and sometimes work as phycocolloids with gelling and viscosity properties depending on the biopolymer’s structural modifications. Gel formation and thermo-versability are the most critical features of carrageenan. Like all biopolymers, carrageenan undergoes aging and syneresis. Isopropyl alcohol or 2-propyl alcohol is used to precipitate carrageenan from the extraction liquid. Therefore, 2-propanol can be observed in the final samples produced, where this material acts as a surfactant [24].

Carrageenan [25] is a unit of naturally derived polysaccharides extracted from algae. Using established procedures for the acquisition of the carrageenan biopolymer, after algae extraction, polysaccharides are precipitated using ethanol or isopropanol [26,27]. In this way, the seaweed *Furcellaria lumbricalis* can be successfully used as a source for the development of a biodegradable force sensor matrix, in which electrically conductive elements (particles) are encapsulated at evenly distributed distances and approach each other corresponding to the applied force, thus, changing the electrical resistance [28,29].

Therefore, carrageenan, which has a history of use for human purposes, was rediscovered in sensing technologies when the application of rubber-like materials and gels in sensor production became available. In general, conductive hydrogels, as a separate class that changes their electrical properties in response to the applied force, are attractive due to their self-healing, good conductivity, and flexibility [30].

The chemical structure of carrageenan located in the outer cell wall and the intracellular matrix of algae tissue [31] depends on the species of red algae and extraction methods. Carrageenans are linear anionic sulfated polysaccharides or sulfate galactose biopolymers composed of galactose and anhydrous-galactose and are divided into types according to repeating disaccharide units [32]. The major subtypes of carrageenan are kappa, iota, and lambda. Kappa or K-carrageenan forms strong gels, iota or i-carrageenan forms soft gels, and lambda carrageenan does not make a gel. Carrageenan subtypes differ in the number of sulfate groups in the hexose scaffold skeleton of plant molecules and contain some negatively charged sulfate ester groups per disaccharide unit [33].

In some cases, the extraction result may be kappa–iota hybrids of carrageenan. As a hydrophilic colloid, K-carrageenan is popular as a gelling agent in many manufacturing fields and has thermal response behavior as a sol-gel transition and gelation ability [34].

As a biopolymer, carrageenan carries out such properties as an antioxidant, antibacterial agent, anti-coagulant, and immune modulator [33]. The gelling properties of carrageenans depend on the structure of its molecule. One way to gelatinize is to apply polyvinyl alcohol (PVA) to provide the elasticity of the carrageenan biopolymer [35]. As an anionic polysaccharide, carrageenan can absorb the surface lipid droplets at pH3 to pH6 better than other marine polysaccharides. Emulsions of carrageenan with NaCl are stable, have high viscosity, and are applicable for different purposes [36].

The structural properties of carrageenan gels can be tuned by changing the salt concentration by adding KCl and CaCl_2_ to kappa and iota carrageenans, and their mixtures [37]. Different concentrations of salts are necessary for the gelation of ι-carrageenan, κ-carrageenan, and Ca^2+^ ions, causing the stiffer k-carrageenan network induced by K^+^ ion [37]. Morris E.R. [38] investigated the influence on carrageenan and reported that the presence of K^+^, Rg^+^, Cs^+^, and high concentrations of Na^+^ influences gel formation. Smidsrod also investigated the gelling mechanism [39]. The elastic modulus of κ-carrageenan gels with Ca^2+^ was investigated by P. MacArtain [40].

As is known, all polysaccharides as anionic biopolymers interact with water via hydrogen bonding. This means that in a biopolymer, the solvent works only on a hydrogen-bonded network, and this network retains unpolymerized molecules in its structure. The Flory–Huggins (FH) [41] theory describes the behavior of polysaccharides, two-phase systems, solvent separation, etc. Many models in the literature are dedicated to defining the elastic pressure of biopolymers, which, together with osmotic pressure and swelling ratios [42] defining the strength and stability, are named as the main characteristics of such materials. The uniaxial compression can be described by the theory of non-linear elasticity [43,44].

Due to many variations in the structure of carrageenan and its combination with other materials, many reports have been dedicated to carrageenan application in sensors. Tao [45] reported on the tactile biopolymer-based sensor made of carrageenan cross-linked with polyacrylic acid (PAM) as a pyramidal biopolymer with good mechanical properties. Adding metals to biopolymers containing PAM chains enhances the mechanical properties of the biopolymer. Qiang Zheng [46,47] reported about a κ-carrageenan/polyacrylamide biopolymer with remarkable mechanical performance as a strain sensor and good stability by using Zr^4+^ ions, cross-linked with PAM. The k-carrageenan with the nano-TiO_2_–anthocyanin layer is an indicator. On the contrary, the TiO_2_–agar layer works due to the strong adhesion between these two hydrophilic colloid layers [48].

Zhifeng Pan proposed stretchable sensors based on carrageenan for self-healing carrageenan-based sensors [49]. C.M. Costa [50] announced a 3D-printed carrageenan-based sensing device with multi-wall carbon nanotubes. M.M. Khodaei [51] presented a compound based on carrageenan–metformin nanoparticles produced using Fe_3_O_4_ magnetic particles. They mentioned carrageenan as an excellent material for sensors. Youshiyuki Nishio investigated the nano incorporation of iron oxides as magnetic compounds [52]. Chang [53] presented the successful application of carrageenan as a resistive switching layer for resistive memory devices. It is necessary to note that the composition of carrageenan with Fe_2_O_3_ nanoparticles was only mentioned as a gas sensor for medical purposes and investigated by analyzing carrageenan as a biological study object [54].

Marine polysaccharides have wide applications in industry, and there are reports of various development routes for this biopolymer. The transformation of the enchanted copolymerization of the K-carrageenan structure by microwave irradiation in aqueous medium results in polymers with enchanted characteristics [38,55]. Carrageenan is commonly applied as an emulsifier, stabilizer, thickener, and gelation agent in cosmetics and foods around the world. However, in the European Union (EU), its use in the food industry is restricted by legal regulations. However, carrageenan was treated as an antiviral material that could be used for medical purposes [33]. The antivirus feature of carrageenans is useful in designing products for natural environments.

### 2.3. The Role of Iron (III) Oxide in the Force Sensor Structure

Iron (III) oxide, with good semiconductor properties, is gaining broader applications and is attractive because of the properties of this material: mechanical, magnetic, catalytic, sorption, semiconductor, thermal, electrical, and capillary [56]. Currently, iron (III) oxide synthesis using microbial and plant extracts has already been achieved. It is an efficient and inexpensive process. Therefore, iron (III) oxide is favored as a green chemistry method. Therefore, using iron (III) oxide in combination with seaweed-derived carrageenan should be relevant for green course solutions [57]. Carrageenan, composed with Fe_2_O_3_ as super-porous matrices with spherical morphology, is applicable for high-flux magnetic field extended bed adsorption [58].

The manufacturing of flexible compression sensors usually has the challenge of connecting working-sensitive material to the electronic parts of a sensor. One of the important procedures in force sensor manufacturing is connecting wires or contacts to the working part of the sensor. Copper foil is usually offered for the electrical connection to assemble the sensor [59]. The alumina wire as a connector is well-studied also. The adsorption of carrageenan on alumina increased in the presence of surfactants [60], and this characteristic must influence the quality of the contact in the sensor. The adsorption on the surface is a complex process and is not fully understood yet [61].

It is important to note that the field of biodegradable force sensors is an active area of research, and advancements are being made to explore new materials, fabrication techniques, and applications. The reports mentioned above highlight the potential of utilizing biodegradable materials for force sensing while minimizing the environmental impact.

## 3. Materials and Methods

### 3.1. Materials and Devices 

All the solutions were prepared using deionized water. All the chemicals (isopropanol, Fe_2_O_3,_ other metal oxides, and glycerol) were purchased from Sigma-Aldrich (Taufkirchen, Germany). All the main materials, such as aluminum foil, transparent plastic plates, etc., were obtained according to the description of the internal procedures of the laboratories. The carrageenan was prepared from seaweed *Furcellaria lumbricalis* in the scientific lab of Liepaja University, Latvia [24,62,63].

The micrographs were made by the custom-made robotic micro-positioning system operated by open-source Linux CNC 2.8.4 and equipped with a digital microscope consisting of a camera with a SONY IMX334 1/1.8-inch sensor (Sony, Tokyo, Japan) and monocular objective with zoom capabilities up to 400 times.

The determination of the mechanical properties of the prepared material samples was performed experimentally by using the computer-controlled tension–compression machine Mecmesim *MultiTest 2.5-i* (Mecmesim Limited, Slinfold, West Sussex, UK, maximum load: 2500 N; maximum sample diameter: 134 mm; load sensor measurement error: ± 0.1%; speed range: 1–1000 mm/min). The testing machine was controlled by the software “Emperor v1.18” (Mecmesin Limited, Slinfold, West Sussex, UK). In our case, 25 N and 1000 N load cells were mounted on the testing machine gripper and used for the experimental research. The resistance measurement was performed using a UNI-T UT55 Multimeter; the measurement accuracy fits within ±2%.

Micrographs of the sample cross-sections were obtained by using a scanning electron microscope (SEM) at Liepaja University, Latvia, using a Hitachi TM3000 tabletop microscope (Hitachi, Tokyo, Japan).

### 3.2. Sample Preparation

The carrageenan gel extracted from seaweeds and prepared for the next steps was stored at 9 °C. The preparation of the biopolymer was performed in the following order.

The carrageenan gel was heated in a 70 °C temperature water bath. Before the experimental procedures, isopropanol was stored at a temperature of −18 °C. Cold isopropanol was suddenly poured into the same amount of hot carrageenan gel solution in a ratio of one part of solvent to one part of the gel and stirred vigorously until a stiff biopolymer had formed. The polymerization process begins immediately after the first drops of isopropanol contact the carrageenan gel. The end of the polymerization process is evaluated visually. The detailed procedure of the biopolymer preparation is given by Zaimis [62]. The prepared biopolymer with the water–isopropanol mixture was tightly closed and stored at 9 °C. After, the prepared mass from the carrageenan gel was filtered through a sieve to separate the liquid phase from the biopolymer. The produced mass of biopolymer shows thread-like disordered segments with chaotic connections. The biopolymer mass separated from the liquid was mixed with selected additives (metal oxides) and placed in a container to form a film approximately 2 mm thick.

After the initial investigation with different metal oxides, the Fe_2_O_3_ was selected. Based on the newly obtained experimental data from our research group, the literature review, and our co-authors’ [64] previous publications, we selected iron oxide as a well-examined environmentally friendly material, not included in active industrial applications. The biopolymer mixed with Fe_2_O_3_ was heated again to melt and gently mixed, enriched with the different amounts of glycerol to avoid complete drying. Thus, the product’s elasticity was preserved most ecologically, without the additional hazardous materials, such as PVA glue or similar. The carrageenan biopolymer was mixed with Fe_2_O_3_ (from 1% to 5%), as the stable biopolymer structure was not forming at higher oxide concentrations. It was experimentally determined that samples containing approximately 1.8% Fe_2_O_3_ in the carrageenan biopolymer showed better mechanical properties (resistance to tearing, scratching, stretching) in the initial assessment and were selected as a composition suitable for the force sensor. The prepared product was transferred into a plastic Petri dish for drying at room temperature. The samples prepared in this way are elastic and flexible. The samples without the glycerol dried out and lost their flexibility within a week if placed in an open area.

Similar samples were prepared in two laboratories in Latvia and Lithuania to compare results and to check the correctness of the preparation protocol. For further experiments, samples with 10%, 15%, and 18% of the glycerol were selected. The prepared samples were stored at ambient temperature in a container protected from moisture loss. The size of the samples selected for the force experiments was approximately 700 mm^2^.

The prepared samples were investigated by optical microscope and SEM to understand the distribution of Fe_2_O_3_ in the biopolymer. An optical microscope was used to research the sample surface, and SEM was used to analyze the cross-section of the prepared samples.

The aging processes of the prepared samples were assessed by storing some of them at room temperature for more than two months and, after that, investigating their properties in a controlled compression bench as the other samples.

### 3.3. Experimental Section

**Figure 1:**
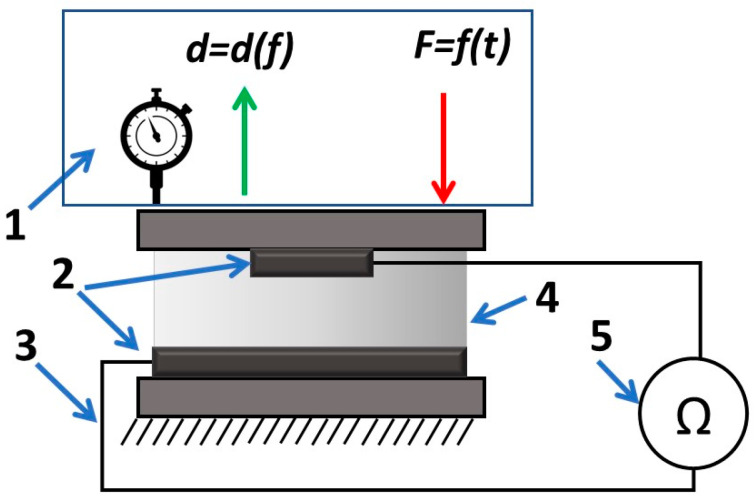
Schematic representation of the experimental setup. 1—mechanical force load system; 2—electrodes; 3—wires; 4—sample; 5—multimeter.

The prepared material samples were placed into a special holder from a transparent 0.3 mm thick plastic film (Figure 1) to test the mechanical and electrical properties. The plastic holder is a base for fixing 10 mm width, 100 mm length, and 0.2 mm thickness electrodes made from aluminum foil. The electrodes were placed across the sensitive material and rotated by a 90-degree angle relative to each other. Therefore, after placing the produced biopolymer between them, a prototype of a force sensor with a sensitive area of 100 mm^2^ at the electrode’s intersection point was formed. The mentioned plastic holder also acted as a protective coating, sealing the biopolymer and protecting it from environmental impact. The used plastic film is relatively thin and much stiffer than the sample material; therefore, its effects on the material’s mechanical properties could be neglected.

The electrodes are made from aluminum since the aluminum and carrageenan interaction is well-studied, and it is known that any phenomenon in the pair of these materials could affect the results of the electrical characteristic measurements.

The measurements of the samples’ mechanical characteristics were complemented with parallel resistance measurements (Figure 1). The samples located in the holder were placed into the tension–compression machine, and, at the same time, electrodes were connected to a resistance measuring device.

At least three samples of each concentration of glycerol (10%, 15%, 18%) were tested by compressing them at a 1 mm/min speed while the load reached 500 N. After removing the compressing force, the relaxation time was registered by observing material resistance variation with a multimeter. The experiment and equipment were filmed using a smartphone to synchronize the data obtained from the multimeter and tension–compression machine.

## 4. Results 

This section provides a set of experimental results obtained by researching the characteristics of pure carrageenan and later combining it with iron (III) oxide and glycerol.

Initially, the characteristics of the pure carrageenan liquid were defined. The ionic conductivity of the prepared pure carrageenan liquid gel is 0.27–0.28 mScm^−1^. The resistance of this liquid is from 0.038 MΩ to 1.6 MΩ when dried. The resistance of carrageenan biopolymer liquid with Fe_2_O_3_ is about 0.08 MΩ. The resistance of the dried sample ranges from 1.2 MΩ to 0.5 MΩ according to the amount of Fe_2_O_3_ in the sample.

**Figure 2:**
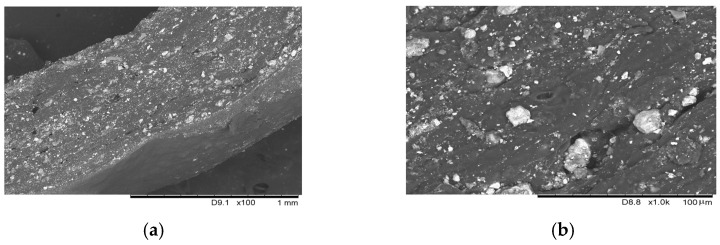
Investigation of the k-carrageenan biopolymer with Fe_2_O_3_ structure by SEM: (**a**) Micrograph of carrageenan biopolymer with Fe_2_O_3_, cross-section. Size 1280 × 1100; DPI = 182.63; Conditions: Vacc = 15.0 kV; Mag = ×100; WD = 10.60 mm, (**b**) Micrograph of carrageenan biopolymer with Fe_2_O_3_ particles, cross-section, Size 1280 × 1100; DPI = 182.65; Conditions: V_acc_ = 15.0 kV; Mag-1.0k; WD = 10.30 mm.

Before evaluating the mechanical and electrical properties, the structure of the prepared samples was investigated under optical and scanning electron microscopes to evaluate the distribution of Fe_2_O_3_ particles in the biopolymer. As is seen from the sample cross-section micrographs (Figure 2), the Fe_2_O_3_ distribution in the film is even enough after evaluating the data. However, it should be noted that the density of Fe_2_O_3_ is higher than the density of the biopolymer. Therefore, Fe_2_O_3_ has the opportunity to settle on the bottom during sample drying.

**Figure 3:**
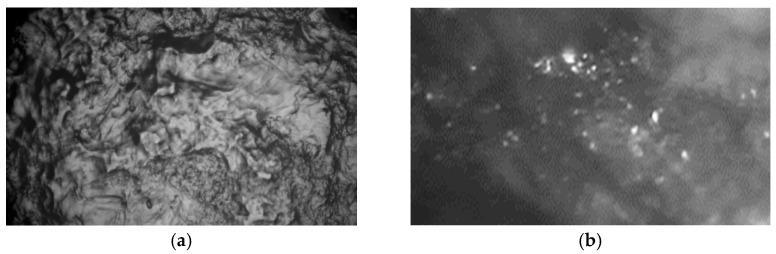
Micrographs of the sample surfaces: (**a**)—pure carrageenan film; (**b**)—carrageenan with iron (III) oxide.

In addition, it is seen that the particles of the chosen iron oxide differ in their size. The larger iron oxide particles are badly embedded into the biopolymer in the micrographs above. There are places in the micrographs with large iron oxide particles surrounded by dark places—air gaps. Such large particles can damage the biopolymer during compression and negatively influence the actual resistance due to air gaps. The sifted particles would improve the quality, but on the other hand, they would also increase the product cost. The micrographs of pure biopolymer and the prepared sample surfaces are presented in Figure 3. It is seen that the iron (III) oxide particles modify the surface of the biopolymer, making it smoother.

**Figure 4:**
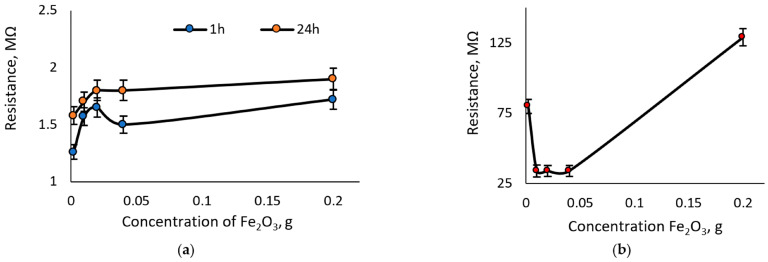
Sensitivity of carrageenan biopolymer samples without glycerol to the environmental impact: (**a**) dependence of the resistance on the concentration of Fe_2_O_3_ in time; (**b**) dependence of resistance vs. concentration of Fe_2_O_3_ after 120 h from sample preparation.

Figure 4 shows the influence of Fe_2_O_3_ concentration on resistance concerning time. It represents material stability and its sensitivity to environmental impact. These characteristics were obtained from new samples before starting the experiments with the load. The freshly prepared carrageenan biopolymer samples with iron (III) oxide show a dependence of the resistance values on metal oxide concentration when the sample is not loaded. From visual evaluation and resistance measurements, it was noticed that environmental humidity could negatively affect the samples. The samples without glycerol, which guarantees the elasticity of this product, are losing relevant properties. As the most stable composition, the samples with 1.6% to 2% Fe_2_O_3_ in the carrageenan biopolymer could be noted.

**Figure 5:**
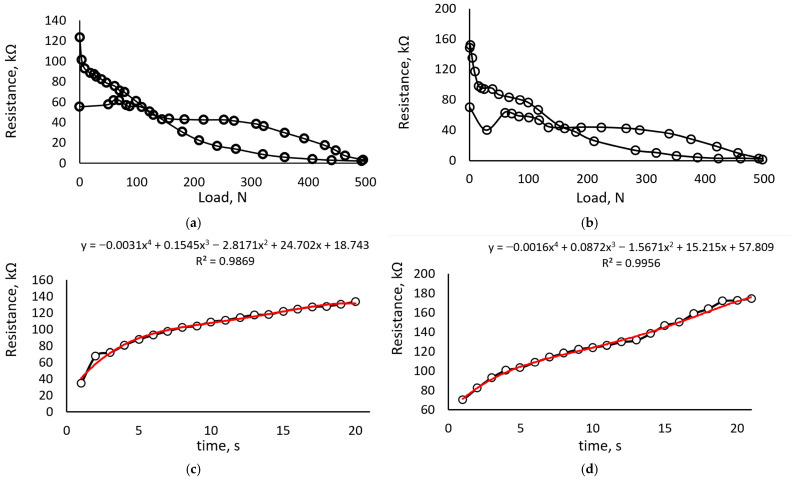
Electrical characteristics of the carrageen–biopolymer composition with Fe_2_O_3_ and 10% glycerol: (**a**) hysteresis of the first loading cycle from 0 to 500 N; (**b**) hysteresis of the fifth loading cycle from 0 to 500 N; (**c**) relaxation of the sample after removing the load, at first experiment and fit curve of polynomial order 4; (**d**) relaxation of the sample after removing the load, at fifth experiment and fit curve of polynomial order 4.

Detailed studies were carried out with a tension–compression machine after validating the suitability of the carrageenan iron oxide and glycerol composition as a sensitive material for force/pressure sensors. The obtained testing results are represented in the figures below. Figure 5 shows the hysteresis and relaxation time of a sample of carrageen-biopolymer composition with Fe_2_O_3_ and 10% glycerol. From Figure 5a,b, significant hysteresis and cycle-dependent characteristic drift, which is typical behavior for such materials, is noticeable. Moreover, non-linear sensitivity is noticeable; the angle of curve inclination drastically changes when the 20 N load value is reached. Therefore, sensor sensitivity characteristics can be divided into two parts: when the biopolymer structure withstands the load without significant deformation (approximately 20 N) and when the carrageenan product starts to deform plastically. Hysteresis as a mechanical behavior in the sensor material occurs due to the high internal friction of the material. This material has very low stiffness and, under high loads, can relax stresses by plastic deformation. Therefore, hysteresis here is unavoidable.

Comparing the relaxation results (Figure 5c,d), obtained by registering the resistance values 20 s after unloading the sample, it is possible to notice similar tendencies. In both cases, the average relaxation time is approximately 17 s, but in the case of relaxation after five cycles, the resistance variation concerning time seems more linear. The rearrangement of the conductive particles and destruction/damage in the biopolymer structure could explain the variation in sensor characteristics regarding the loading cycles.

**Figure 6:**
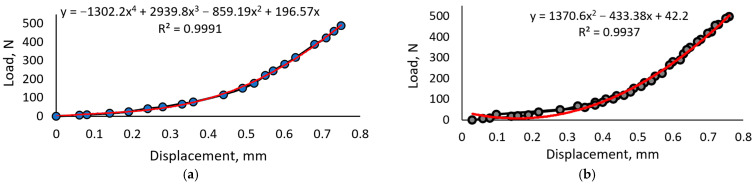
Mechanical characteristics of the carrageen–biopolymer composition with Fe_2_O_3_ and 10% glycerol: (**a**) dependency between load and sensor deformation during the first loading; (**b**) dependency between load and sensor deformation during the fifth loading cycle.

The dependencies between sensor deformation and load are represented in Figure 6. It is seen that the variation in mechanical characteristics is less noticeable compared to electric ones. In the first and fifth loading cycles, the overall deformation almost equals 0.75 mm under a 500 N load. However, the data obtained from the fifth cycle show some minor fluctuations referring to internal changes in the material’s structure, for example, the rearrangement or break-up of the Fe_2_O_3_ particles.

**Figure 7:**
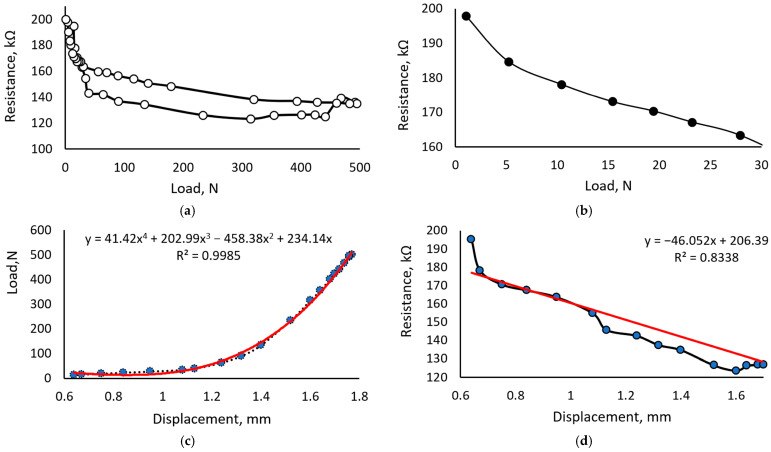
Characteristics of the sample containing 15% of the glycerol: (**a**) hysteresis defined at the fifth loading cycle from 0 to 500 N; (**b**) sensor sensitivity in the range from 0 to 30 N; (**c**) dependency between load and sensor deformation during the fifth loading cycle; (**d**) dependency between resistance and displacement during the fifth loading cycle.

Figure 7 provides the results of the sample prepared with 0.5 mL of glycerol added to 100 g of carrageenan liquid, resulting in a glycerol concentration of 15%.

Figure 7a provides a hysteresis of the sample with 15% glycerol, defined after five loading cycles. The shape of the obtained curve allows us to assume that sensor stiffness is relatively low and internal friction in the material causes significant energy losses when deformed. In addition, one can see that the hysteresis loop is shifted to the side of the higher loads and unevenly distributed in the range of the tested loads. A sudden change in sensor characteristics occurs when the load exceeds 30 N. Therefore, sensitivity in the load range from 0 to 30 N was observed in more detail (Figure 7b), defining that it is significantly better than in the remaining load range.

The dependency between displacement and load (Figure 7c) showed a non-linear relation, while displacement vs. resistance (Figure 7d) was almost linear. Such behavior confirms the hypothesis that material resistance changes mainly due to the variation in contact surface area between conductive particles due to the increasing or decreasing distances between them. Furthermore, the determined characteristics show that samples with 15% glycerol are better for measuring forces up to 3 N, and in case of higher loads, using this sensor only as a binary switch is more reliable.

The results obtained from testing samples with 0.6 mL of glycerol corresponding to the concentration of 18% are provided in Figure 7.

**Figure 8:**
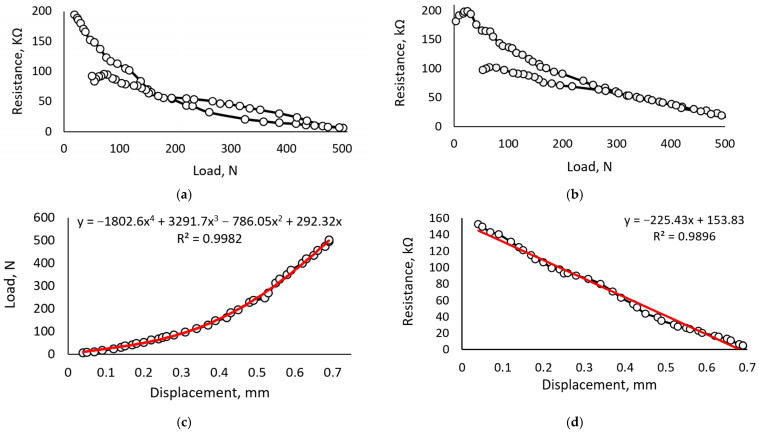

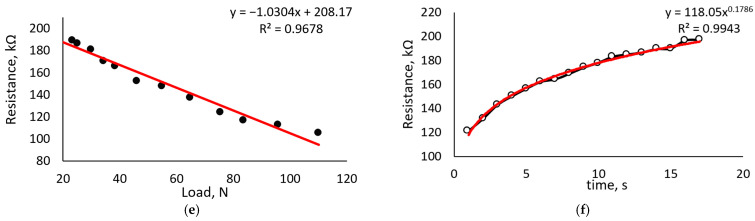
Characteristics of the sample containing 15% of the glycerol: (**a**) hysteresis defined after the first loading cycle; (**b**) hysteresis defined after the fifth loading cycle; (**c**) dependency between load and resistance; (**d**) dependency between displacement and resistance; (**e**) sensor sensitivity in the range from 20 to 100 N; (**f**) relaxation of the sample after removing the load.

The hysteresis of the sample containing 18% glycerol (Figure 8a) is smaller compared to the sample with 15% glycerol. Still, in this case, a higher difference between the sensor response to the decreasing and increasing load is noticeable in the range from 20 to 150 N. After five loading cycles (Figure 8b), hysteresis becomes negligible for loads higher than 250 N but increases for the smaller loads. Such behaviors point to the properties typical for all flexible piezoresistive materials—their characteristics vary concerning the amount and types of the loading cycles. When using contact materials, ensuring electrical transmission depends on the microstructure of the materials [65,66,67], especially in our case, where iron (III) oxide and carrageenan are working in contact with alumina wires.

It is assumed that the absolute value of characteristic shifts could also be affected by the contact interaction features between the aluminum contact and sensor film. The same reason could also explain the slightly unstable behavior observed under loads below 20 N.

The dependency between displacement and load (Figure 8c) shows that the mechanical characteristics of the sample are similar to the sample containing 15% of glycerol. Still, this sample is stiffer, and the dependency between displacement and resistance (Figure 8d) is even more linear. Similarly, the linear part of the sensor sensitivity curve is more significant (Figure 8e). The linear relation between load and resistance exists in the load range from 20 N to 110 N. The relaxation time during which resistance increases to 90% of the initial value for this material composition is approximately 12 s.

In a comparison of all three compositions researched, the best results are shown for the sample containing 18% glycerol. Such results highlight that the glycerol in this biopolymer plays an essential role in retaining moisture, which helps self-heal the biopolymer and protects it from the damage and abrasion caused by iron oxide (III) oxide particles.

## 5. Discussion

The sensitivity and hysteresis characteristics of the tested samples show their different responses to the mechanical load values. The initial resistance of all the tested samples was approximately equal to 200 kΩ. However, when samples were tested with a 500 N load, the resistance of the samples containing 10% and 18% glycerol decreased to 2.3 kΩ and 7.1 kΩ, respectively. In comparison, the resistance of samples containing 15% glycerol remains in the range of 120 kΩ. Furthermore, the sensitivity characteristics of all the tested compositions are partly linear, and two intervals with different sensitivities are noticeable. For the material compositions containing 10% and 15% glycerol, a sudden sensitivity change was observed under the 20 N and 30 N loads. The composition containing 18% glycerol showed a smooth response, and a sensitivity change appeared approximately at a 160 N load in the case of the first load cycle. In contrast, it offered an almost linear response after the five loading cycles. Such results show that the glycerol concentration could have an impact on the material and the resistance between aluminum contacts and the biopolymer.

The evaluation of the deformation of the materials under the loads also revealed some unambiguous impact of the glycerol concentration on the stiffness of the material. The most exceptional results were provided in samples containing 15% glycerol; deformation under the 500 N load reached 1.75 mm, while samples containing 10% and 18% deformed 0.75 mm and 0.66 mm, respectively. The curves representing the resistance dependence on displacement were fitted using the polynomial fourth-order function. The fitting results (R-squared more than 0.998) proved that the mechanical behavior of all the compositions tested corresponded to the polynomial model, applicable when the energy of the deformation was continuously differentiable several times with respect to three strain invariants for compressible materials and two invariants for incompressible ones [43]. The polynomial model fits well to describe the material characteristics in the finite element method (FEM) and corresponds to the recommendation provided in [68], where various functional forms and math expressions were proposed to describe invariants for deformation tensors and principal stretches, and the polynomial model was presented as the most suitable for hyper-elastic materials.

The analysis of the dependencies between material deformation and resistance showed that resistance is linearly proportional to deformation, and the glycerol concentration does not have a significant impact on these characteristics. However, it affects the resistance between the biopolymer surface and the electrodes and could introduce some non-linearities, as was observed in the testing samples containing 15% glycerol.

The measurements of the material relaxation parameters showed typical behavior for piezoresistive elastic material. The average relaxation time for all the compositions tested was approximately 17 s. That is how long it took for the resistance to stop increasing after five loading cycles. However, the speed of the resistance change varied during the relaxation process. The highest values were observed during the first four seconds. The sample containing 18% of the glycerol achieved a resistance increase speed equal to 10 kΩ/s, and the sample with 15% of the glycerol, correspondingly, 7.5 kΩ/s. In the remaining part of the relaxation cycle, all samples showed a similar resistance increase speed, approximately equal to 6 kΩ/s. The relatively long relaxation process can be explained by the excessive impact of the load on the biopolymer structure; the variation in measurement values between cycles shows that the structure requires some time to recover. Furthermore, it should be noted that all compositions were tested under loads of up to 500 N to achieve plastic deformation of the sensor material. However, it should be noted that it is a significant load for such a type of material, and for real applications, the ratio between the maximum load and sensor size must be optimal to avoid the irreversible deformation of the material.

**Figure 9:**
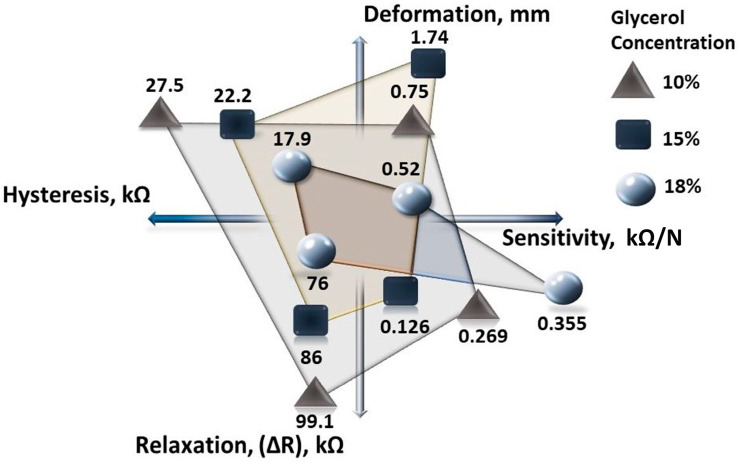
The summarized comparison of the characteristics of the researched carrageenan–iron (III) oxide compositions with different glycerol concentrations.

A systematic comparison of the characteristics of all tested material compositions shows that all of them could be used for force or pressure measurements (Figure 9).

The composition containing 18% glycerol demonstrates the best characteristics of the flexible force or pressure sensor with a sensitive area of 100 mm^2^ capable of operating in the load range from 0 to 500 N. This composition has the highest sensitivity—0.355 kΩ/N, the smallest hysteresis of 17.9 kΩ, and the lowest deformation of 0.52 mm. Consequently, the resistance variation during the relaxation process is also lower.

The analysis of all the experimentally obtained data shows the complicated impact of the glycerol concentration on the materials’ characteristics essential for use in sensing applications. A tricky glycerol impact was noticed in the observance of material deformation. The highest deformation was observed in the composition containing 15% glycerol, while other characteristics showed average values between 10% and 18% concentrations. From Figure 9, it appears that a higher concentration of glycerol ensures better characteristics. However, it is necessary to note that attempts to produce samples with concentrations higher than 18% were unsuccessful, as no stable film was formed.

## 6. Conclusions

Carrageenan, as a biopolymer with a wide application range and a long history of use for various human needs, including in the food industry, pharmaceutical production, etc., is a safe material in the human environment and could be used in contact with the human body. The abundance of carrageenan in the environment and its resistance to viruses have already found applications in medicine. However, the possibilities of applying carrageenan more efficiently and sustainably for everyday needs are economically beneficial and correspond to sustainability issues, forcing the replacement of polluting materials by materials that are safer and easier to recycle or reuse. Due to the ecological requirements for electronic products and the reduction of environmental pollution, by many authors, carrageenan is considered to be an excellent material for creating environmentally friendly biodegradable substrates for wearable electronics. Therefore, the capability of using carrageenan as a base material for force sensors was researched.

The results of this research point to these conclusions and future research:

- The iron (III) oxide powder added into the carrageenan ensures the electric conductivity of the solution. However, it affects the cross-linking of biopolymer molecules and causes corresponding changes in its structure. The most promising results are provided by a composition containing 1.8% Fe_2_O_3_.
- The pure composition of carrageenan and Fe_2_O_3_ powder demonstrates the piezoresistive properties, but the material characteristics are extremely sensitive to the humidity level of the material and the environmental impact.
- Glycerol is a suitable material to stabilize the characteristics of carrageenan and Fe_2_O_3_ compositions. However, the glycerol concentration in the solution significantly impacts material electric conductivity elasticity and hysteresis. The most promising is a combination containing 18% glycerol.
- The material composition containing 1.8% Fe_2_O_3_ and 18% glycerol is suitable for producing tactile force or pressure sensors, ensuring a sensitivity of 0.355 kΩ/N in the load range from 0 N to 500 N.
- Varying the concentration of Fe_2_O_3_ and glycerol in the carrageenan makes it possible to optimize sensor characteristics regarding its specific use case while maintaining relatively low manufacturing costs and exploiting its environmentally friendly features.
- The results of the performed research prove the suitability of the tested materials for implementation in sensing applications; however, the main direction for the future is more detailed research on long-term characteristics under various loads and the implementation of data processing algorithms capable of compensating for characteristic drift in a short period.

## Data Availability

The data presented in this study are available on request from the corresponding author.
